# Mutualism and Dispersal Heterogeneity Shape Stability, Biodiversity, and Structure of Theoretical Plant–Pollinator Meta-Networks

**DOI:** 10.3390/plants14142127

**Published:** 2025-07-10

**Authors:** Chinenye Assumpta Onyeagoziri, Henintsoa Onivola Minoarivelo, Cang Hui

**Affiliations:** 1 African Climate and Development Initiative, University of Cape Town, Cape Town 7701, South Africa; 2 Centre for Invasion Biology, Department of Mathematical Sciences, Stellenbosch University, Stellenbosch 7602, South Africa; 3 International Center for Tropical Agriculture (CIAT), Cape Town 7708, South Africa; 4 Biodiversity Informatics Unit, African Institute for Mathematical Sciences, Cape Town 7945, South Africa; 5 National Institute for Theoretical and Computational Sciences, Stellenbosch 7602, South Africa

**Keywords:** mutualistic interactions, meta-network dynamics, dispersal, stability, abundance, unevenness, compositional similarity, nestedness, eigenvalues, spatial heterogeneity

## Abstract

Mutualistic interactions are crucial to the structure and functioning of ecological communities, playing a vital role in maintaining biodiversity amidst environmental perturbations. In studies of meta-networks, which are groups of local networks connected by dispersal, most research has focused on the effect of dispersal on interaction networks of competition and predation, without much attention given to mutualistic interactions. Consequently, the role of different dispersal rates (between local networks and across species) in stability and network structures is not well understood. We present a competition–mutualism model for meta-networks where mutualistic interactions follow a type II functional response, to investigate stability and species abundance dynamics under varying dispersal scenarios. We specifically assess the impact of mutualism and dispersal heterogeneity, both between local networks and across species, on the structure and stability of meta-networks. We find that mutualistic meta-networks exhibit greater stability, higher total abundance, lower species unevenness, and greater nestedness compared to meta-networks with only competition interactions. Although dispersal heterogeneity across species exerts some influence, dispersal heterogeneity between local networks mainly drives the patterns observed: it reduces total abundance, increases unevenness, and diminishes compositional similarity across the meta-network. These results highlight the pivotal role of both mutualism and spatial dispersal structure in shaping ecological networks. Our work advances understanding of how mutualistic interactions and dispersal dynamics interact to influence biodiversity and stability in complex ecosystems.

## 1. Introduction

Biodiversity maintenance in species-rich communities, under persistent perturbations in the ecosystem, remains a long-standing quest in ecology [1,2,3]. One explanation lies in the complex ecological networks formed by interacting species, particularly mutualistic networks such as those between flowering plants and the pollinators that sustain seed production in plant communities [4] and dictate adaptive foraging effort allocation among pollinators [5,6]. These species rarely coexist in isolation, but rather form networks whose structure and function are essential to ecosystem stability [7,8,9]. However, habitat fragmentation and climate change have led to the spatial fragmentation of these networks into discrete local networks/communities. To preserve such fragmented local networks, the dispersal of propagules between local networks is needed, which could rescue the local population from extinction [10].

**Figure 1:**
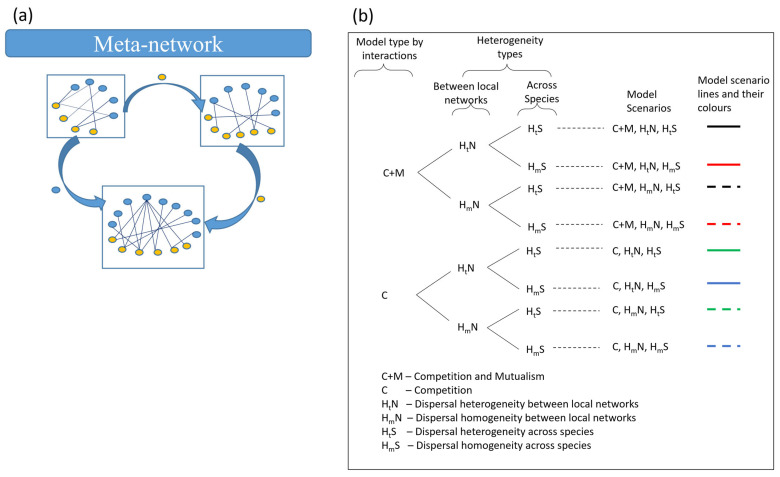
Schematic representation of (**a**) the meta-network and (**b**) the different model scenarios, illustrating models incorporating varying levels of dispersal heterogeneity and interaction types: dispersal heterogeneity between local networks (H_t_ N), dispersal homogeneity between local networks (H_m_ N), dispersal heterogeneity across species (H_t_ S) within a local network, dispersal homogeneity across species (H_m_ S) within a local network, and the competition–mutualism model (C+M) and competition-only model (C). The model scenarios correspond to the coloured solid and dashed lines, which are consistently used across Figure 2, Figure 3, Figure 4, Figure 5, Figure 6, Figure 7 and Figure 8.

When dispersal links multiple such local networks across a landscape, a meta-network is formed (Figure 1a). Specifically, a meta-network can be defined as a group of spatially structured local networks that are linked by the dispersal of species [10]. For example, pollinators may roam seasonally between floral communities and thus couple local plant–pollinator networks [11]. Similarly, animals may move between local networks in response to shifting resource availability, thereby influencing the abundance and dynamics of resource species [11]. Seeds and propagules of plants can also be dispersed between community patches by multiple vectors (e.g., animals and wind; [12]). In essence, the meta-network concept integrates the spatial and temporal dynamics of a group of local networks/communities and provides a means to examine how dispersal and local biotic interactions jointly shape biodiversity and ecosystem resilience [8].

Mutualistic interactions such as pollination are widespread, and play a crucial role in ecosystem functioning [7], the maintenance of biodiversity [13], and the support of human food security [14]. In natural ecosystems, mutualistic interactions play a significant role in determining community structures and species abundances [15,16,17]. For instance, mutualism has been argued to be capable of boosting the abundance of involved species [18], but it imposes a negative effect on the stability of local networks [19], which could lead to alternative community regimes [20]. In contrast, some also found that increasing mutualistic interactions could actually lead to more stable networks [21]. Most theoretical research in meta-networks/metacommunities, however, has been focused on randomly assembled networks with antagonistic interactions (e.g., predation in food webs) [22,23,24,25], with a clear lack of research efforts in the role of mutualistic interactions in meta-networks. As such, the role of mutualism in the stability of a meta-network has yet to be fully explored. Hence, studying mutualistic meta-networks can be insightful for explaining the structure and function of an ecosystem.

Additionally, the role of dispersal on ecological networks has been proposed to preserve biodiversity and the stable coexistence of species by linking local networks [26]. The dispersal process can enhance overall system stability, as demonstrated in studies using both local stability analysis (e.g., linearisation of the Jacobian matrix) and persistence or viability analysis of species abundance [24,27]. Importantly, local stability tends to improve at intermediate dispersal rates [24]. Even when stability is described by other metrics such as robustness, stability was found to be maximised at intermediate dispersal rates [25,28].

Dispersal also contributes to increased species abundance and persistence in consumer–resource systems, especially under source–sink dynamics [29]. Moreover, it influences community structure by affecting patterns of unevenness in species abundance and compositional similarity between local networks. Unevenness in a network reflects the extent to which species abundances differ within a local network or over the entire meta-network, while compositional similarity compares the relative abundances of species between networks, showing how closely local communities resemble one another [30]. For example, high dispersal rates can increase community evenness and lessen the chance of extinction of rare species [31], while moderate density-dependent dispersal rates can enhance large-scale spatial synchrony in species abundance [32,33]. In an experiment involving two active bacterial communities, dispersal was found to increase the similarity of the two communities [34]. Interestingly, dispersal was found to increase evenness in one community, but not the other community, although both were simultaneously experiencing a disturbance [34].

Beyond species-specific effects, dispersal also affects the structural properties of meta-networks, such as nestedness and modularity. Regarding the nestedness pattern in meta-networks, where species-poor sites contain proper subsets of the assemblages of species-rich sites, dispersal was found to reduce the nestedness in a meta-network of mesocosms exposed to varying nutrient levels. However, when the nutrient levels are the same, nestedness remained low and was found to be insensitive to the increase in dispersal rate [35]. By contrast, in another study incorporating empirical and theoretical networks, modularity was reported to decrease with the increase in dispersal rates [36]. In this context, modularity was referred to as the degree to which some local networks have a higher flow of individuals among themselves than with other local networks, forming several well-connected movement hubs. These findings highlight the context-dependent effects of dispersal on both species-level and network-level ecological dynamics.

Importantly, dispersal rates of species can vary substantially between local networks, often resulting in source–sink dynamics, where some local networks act as sources of propagules and others as sinks [37]. While some theoretical studies have explored the role of dispersal in meta-networks, most have assumed homogeneous dispersal rates between local networks [24,38]. These studies have shown that dispersal can enhance the coupling of local networks and influence the overall structure of the meta-network [24,25,38,39,40]. However, in a more realistic case, where dispersal rates are spatially varying and context-dependent, we should anticipate dispersal heterogeneity [23,41]. Notably, such dispersal heterogeneity between local networks was found to destabilise a consumer–resource meta-network [23]. Yet, the broader implication of dispersal heterogeneity, particularly its effects on species-level properties such as unevenness and compositional similarity, and network-level features such as nestedness and modularity, remains poorly understood. In this study, we address this gap by systematically investigating how dispersal heterogeneity between local networks and across species shapes the dynamics and structure of mutualistic meta-networks (Figure 1).

Building on classical competition–mutualism models [15,42], we develop a competition–mutualistic meta-network model that captures the dynamics of species interactions across spatially structured communities. Specifically, we incorporate the mutualistic interactions using a type II functional response to reflect saturation in the benefits obtained from mutualistic partners, while dispersal rates are represented by a linear term of density [24]. With this modelling framework, we systematically explore the effects of mutualism and dispersal heterogeneity, both between local networks and across species, on key ecological properties, including stability, total abundance at equilibrium, species unevenness, compositional similarity, nestedness, and modularity, at both local and meta-network levels.

In this study, we aim to (i) assess how mutualistic interactions, compared to competition-only interactions, influence the structure, biodiversity, and stability of meta-networks; and (ii) investigate how dispersal heterogeneity, both between local networks and across species, affects stability, biodiversity, and network structures. We show that while mutualism has a positive effect on community stability, the effects of dispersal heterogeneity are context-dependent and influenced by the interaction type and spatial configuration of the meta-network. These objectives address critical gaps in understanding the combined roles of mutualism and dispersal in shaping ecological networks.

## 2. Results

Here we explore how mutualistic strength and dispersal heterogeneity influence the stability and persistence of ecological communities. We developed a spatially implicit meta-network model comprising two interacting groups, including plants and pollinators, that are distributed across multiple mutualistic local networks. Dispersal processes link the species across space (see more details in “Section 4”) and, together with varying interaction and heterogeneity types, we have eight model scenarios (Figure 1b).

### 2.1. Stability

**Figure 2:**
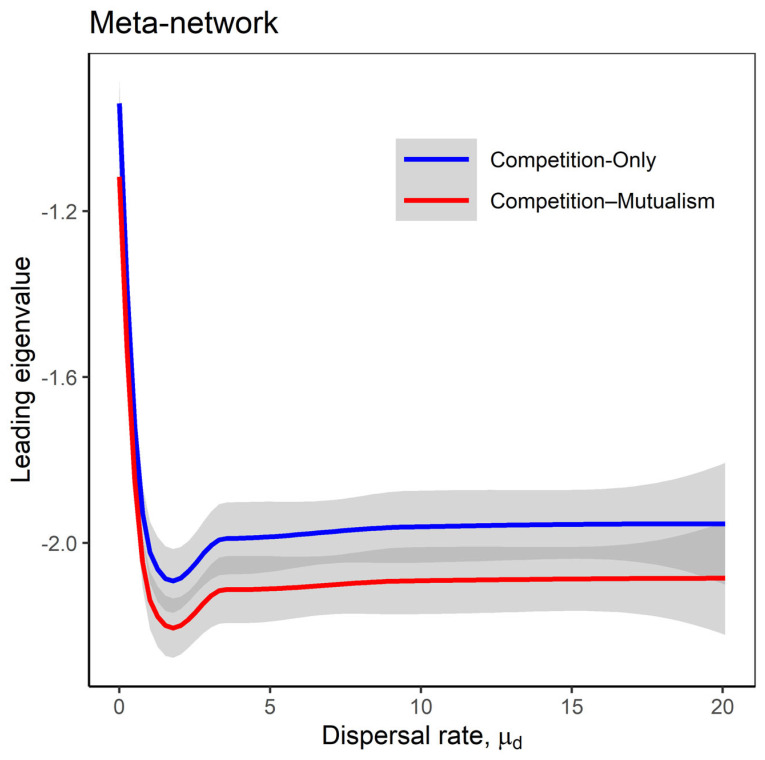
The leading eigenvalues of the meta-network as the dispersal rate $\mu_{d}$ increases. The lines represent the Loess regression of the leading eigenvalues, with shaded grey areas indicating the standard error. The simulated scenarios consider the homogeneous dispersal rate between the local networks and across species, under both the competition-only (C) and competition–mutualism (C+M) models. Fixed parameters are as follows: *M* = 30; *N* = 20; *C* = 0.2; *m* = −1; *μ*_1_ = 0; *σ*_1_ = 0.05; *μ*_2_ = 0; *σ*_2_ = 0.05; *S* = *M* + *N*; *n* = 10; *σ_d_* = 0.01.

**Figure 3:**
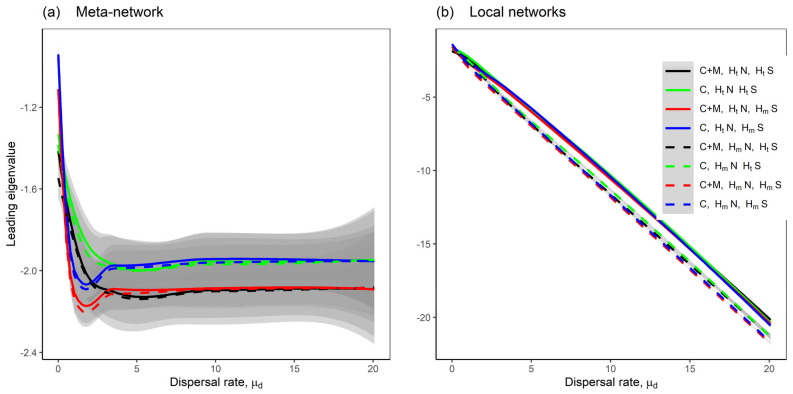
The leading eigenvalues of the networks as the dispersal rate $\mu_{d}$ increases. The lines represent the Loess regression of the leading eigenvalues, with shaded grey areas indicating the standard error; (**a**) shows the meta-network, while (**b**) shows the local networks, where the dispersal rate represents the average over ten local networks. Eight model scenarios were considered; the solid lines are the scenarios with heterogeneous dispersal rates between local networks; the dashed lines are the scenarios with homogeneous dispersal rates between local networks; the black and red lines are the scenarios with the competition–mutualism model; the green and blue lines are the scenarios with the competition-only model; the scenarios with heterogeneous dispersal rates across species where $\sigma_{d}=0.5$ are shown in black and green lines, while the scenarios with homogeneous dispersal rates across species where $\sigma_{d}=0.01$ are shown in red and blue lines. A description of the scenarios is provided in Figure 1b. Fixed parameters are as follows: *M* = 30; *N* = 20; *C* = 0.2; *m* = −1; *μ*_1_ = 0; *σ*_1_ = 0.05; *μ*_2_ = 0; *σ*_2_ = 0.05; *S* = *M* + *N*; *n* = 10.

Our results indicate that mutualistic interactions enhance the stability of meta-networks compared to meta-networks with competition-only interactions, particularly under conditions of homogeneous dispersal rates both between local networks and across species (Figure 2; also see Supplementary Figure S1 when comparing random, competition-only, competition–mutualism, and mutualism-only interaction types using the model in [24]). Across all model scenarios, the stability of the meta-networks increased with higher dispersal rates but plateaued at a threshold where further increases in dispersal rate did not enhance stability (Figure 3a). In contrast, local network stability continued to increase linearly with dispersal (Figure 3b). This pattern arises because, as dispersal increases, the diagonal elements of the Jacobian matrices (which represent intraspecific feedback) become increasingly negative (Supplementary Figure S2b–d), stabilising local dynamics.

### 2.2. Total Abundance

**Figure 4:**
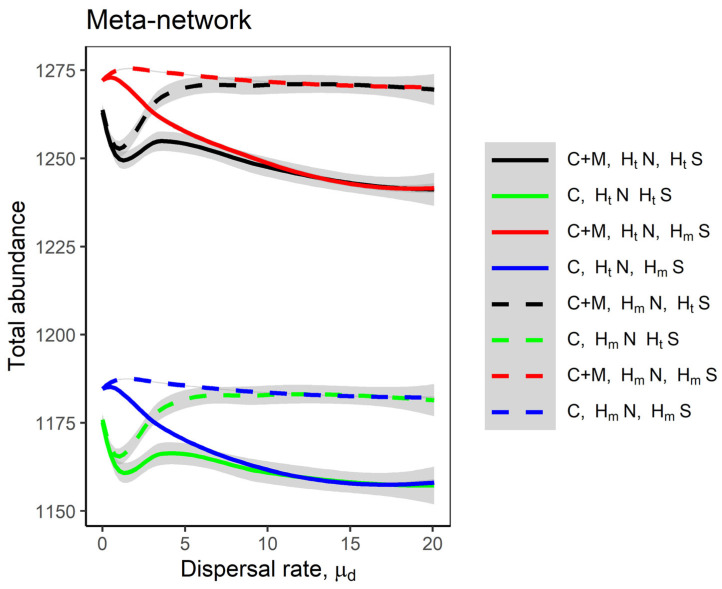
The total abundance of species in the meta-networks as the dispersal rate $\mu_{d}$ increases. The lines represent the Loess regression of the total abundance, with shaded grey areas indicating the standard error. The same eight model scenarios were considered as in Figure 3. A description of the scenarios is provided in Figure 1b. Fixed parameters are as follows: *M* = 30; *N* = 20; *C* = 0.2; *m* = −1; *μ*_1_ = 0; *σ*_1_ = 0.05; *μ*_2_ = 0; *σ*_2_ = 0.05; *S* = *M* + *N*; *n* = 10.

Mutualistic interactions also substantially increase the total species abundance in the meta-network, especially at higher dispersal rates (Figure 4, black and red lines). In addition, the effects of dispersal heterogeneity were pronounced; specifically, heterogeneous dispersal between local networks decreased the total abundance of species in the meta-network (Figure 4, red and black solid lines for competition–mutualism models; blue and green solid lines for competition-only models). Homogeneous dispersal between local networks led to different outcomes depending on species-level dispersal variability: (i) when species had heterogeneous dispersal rates, total abundance increased (Figure 4, black dashed line for competition–mutualism model; green dashed line for competition-only model) and (ii) when species had homogeneous dispersal rates, total abundance remained unchanged (Figure 4, red dashed line for competition–mutualism model; blue dashed line for competition-only model). Thus, mutualism and homogeneous dispersal rates across local networks together maximise meta-network abundance, while dispersal heterogeneity between local networks reduces it.

### 2.3. Unevenness

**Figure 5:**
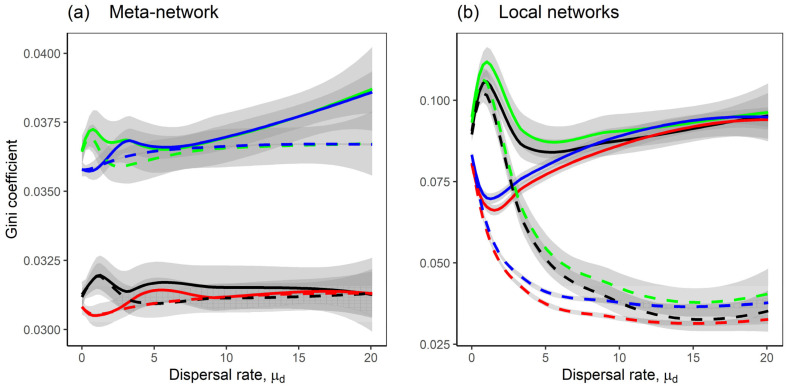
The Gini coefficient of the networks as the dispersal rate $\mu_{d}$ increases. The lines represent the Loess regression of the Gini coefficient, with shaded grey areas indicating the standard error; (**a**) shows inequality in species abundance at the level of the meta-network, while (**b**) shows average inequality within ten local networks. Eight model scenarios were considered; the solid lines are the scenarios with heterogeneous dispersal rates between local networks; the dashed lines are the scenarios with homogeneous dispersal rates between local networks; the black and red lines are the scenarios with the competition–mutualism model; the green and blue lines are the scenarios with the competition-only model; the scenarios with heterogeneous dispersal rates across species where $\sigma_{d}=0.5$ are shown in black and green lines, while the scenarios with homogeneous dispersal rates across species where $\sigma_{d}=0.01$ are shown in red and blue lines. A description of the scenarios is provided in Figure 1b. Fixed parameters are as follows: *M* = 30; *N* = 20; *C* = 0.2; *m* = −1; *μ*_1_ = 0; *σ*_1_ = 0.05; *μ*_2_ = 0; *σ*_2_ = 0.05; *S* = *M* + *N*; *n* = 10.

The competition–mutualism meta-networks were generally less uneven in species abundance than the competition-only meta-networks (Figure 5a, black and red lines for competition–mutualism). Dispersal further modulated species abundance unevenness depending on its heterogeneity: (i) homogeneous dispersal between local networks reduced unevenness in both meta-network and local networks (Figure 5a, blue and green dashed lines, Figure 5b, all dashed lines), leading to more equitable distribution of species abundance. In contrast, (ii) heterogeneous dispersal between local networks increased unevenness in species abundance in the local networks (Figure 5b, all solid lines) and in the meta-networks (Figure 5a, blue and green solid lines—competition-only models). As such, dispersal heterogeneity introduces inequality in species abundance distribution, while mutualism helps mitigate unevenness.

### 2.4. Compositional Similarity

**Figure 6:**
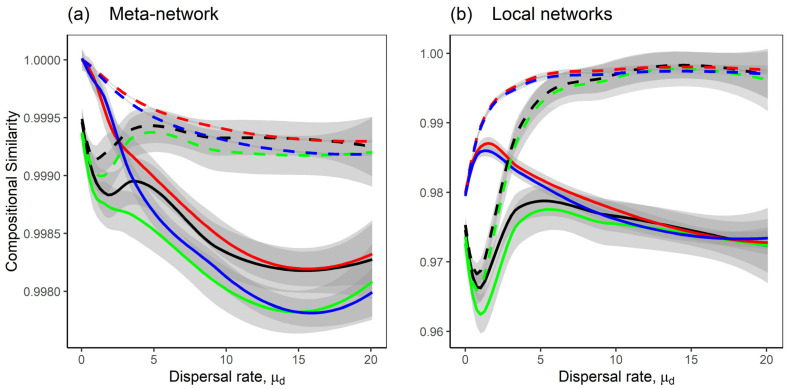
The compositional similarity of the networks as the dispersal rate, $\mu_{d}$ increases. The lines represent the Loess regression of the compositional similarity, with shaded grey areas indicating the standard error; (**a**) shows temporal similarity between meta-networks, with versus without dispersal, while (**b**) shows average spatial similarity between local networks. Eight model scenarios were considered; the solid lines are the scenarios with heterogeneous dispersal rates between local networks; the dashed lines are the scenarios with homogeneous dispersal rates between local networks; the black and red lines are the scenarios with the competition–mutualism model; the green and blue lines are the scenarios with the competition-only model; the scenarios with heterogeneous dispersal rates across species where $\sigma_{d}=0.5$ are shown in black and green lines, while the scenarios with homogeneous dispersal rates across species where $\sigma_{d}=0.01$ are shown in red and blue lines. A description of the scenarios is provided in Figure 1b. Fixed parameters are as follows: *M* = 30; *N* = 20; *C* = 0.2; *m* = −1; *μ*_1_ = 0; *σ*_1_ = 0.05; *μ*_2_ = 0; *σ*_2_ = 0.05; *S* = *M* + *N*; *n* = 10.

Dispersal strongly influenced both spatial similarity (between local networks) and temporal similarity (with versus without dispersal implemented). Temporal similarity between meta-networks was high, greater than 0.9, indicating that species composition converged with dispersal (Figure 6a). Temporal similarity was stronger under homogeneous dispersal between local networks (Figure 6a, dashed lines) than under heterogeneous dispersal (Figure 6a, solid lines). Spatial similarity between local networks was also high (>0.96), with more similarity under homogeneous dispersal and less under heterogeneous dispersal (Figure 6b). This suggests that heterogeneity in dispersal rates between local networks amplifies spatial turnover in species composition (Figure 6b, solid lines). Interestingly, mutualism had little effect on compositional similarity; meta-network and local networks with mutualism converged to equilibrium points similar to those without mutualism, for both homogeneous and heterogeneous dispersal scenarios.

### 2.5. Nestedness and Modularity

**Figure 7:**
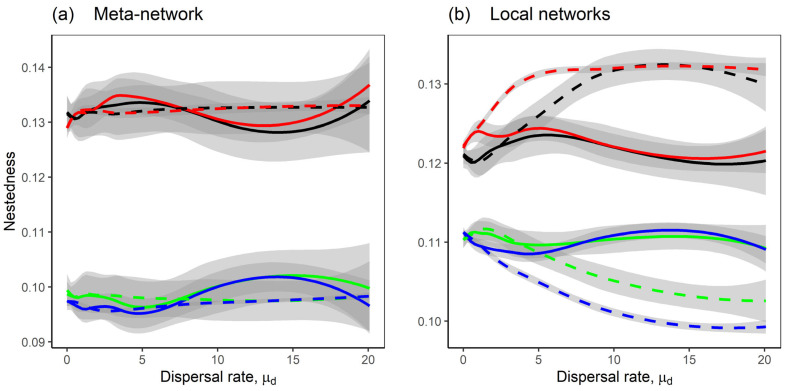
The nestedness of the networks as the dispersal rate $\mu_{d}$ increases. The lines represent the Loess regression of the nestedness, with shaded grey areas indicating the standard error; (**a**) shows nestedness of meta-networks, while (**b**) shows average nestedness of local networks. Eight model scenarios were considered; the solid lines are the scenarios with heterogeneous dispersal rates between local networks; the dashed lines are the scenarios with homogeneous dispersal rates between local networks; the black and red lines are the scenarios with the competition–mutualism model; the green and blue lines are the scenarios with the competition-only model; the scenarios with heterogeneous dispersal rates across species where $\sigma_{d}=0.5$ are shown in black and green lines, while the scenarios with homogeneous dispersal rates across species where $\sigma_{d}=0.01$ are shown in red and blue lines. A description of the scenarios is provided in Figure 1b. Fixed parameters are as follows: *M* = 30; *N* = 20; *C* = 0.2; *m* = −1; *μ*_1_ = 0; *σ*_1_ = 0.05; *μ*_2_ = 0; *σ*_2_ = 0.05; *S* = *M* + *N*; *n* = 10.

**Figure 8:**
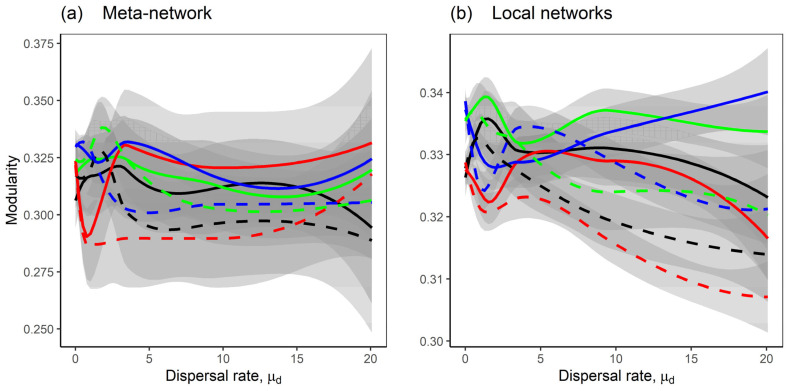
The modularity of the networks as the dispersal rate $\mu_{d}$ increases. The lines represent the Loess regression of the modularity, with shaded grey areas indicating the standard error; (**a**) shows modularity of the meta-network, while (**b**) shows average modularity of the local networks. Eight model scenarios were considered; the solid lines are the scenarios with heterogeneous dispersal rates between local networks; the dashed lines are the scenarios with homogeneous dispersal rates between local networks; the black and red lines are the scenarios with the competition–mutualism model; the green and blue lines are the scenarios with the competition-only model; the scenarios with heterogeneous dispersal rates across species where $\sigma_{d}=0.5$ are shown in black and green lines, while the scenarios with homogeneous dispersal rates across species where $\sigma_{d}=0.01$ are shown in red and blue lines. A description of the scenarios is provided in Figure 1b. Fixed parameters are as follows: *M* = 30; *N* = 20; *C* = 0.2; *m* = −1; *μ*_1_ = 0; *σ*_1_ = 0.05; *μ*_2_ = 0; *σ*_2_ = 0.05; *S* = *M* + *N*; *n* = 10.

Mutualism significantly enhanced the nestedness of both meta-networks and local networks, compared to competition-only interactions (Figure 7, black and red lines). For the competition–mutualism model, homogeneous dispersal between local networks promoted higher nestedness than heterogeneous dispersal (Figure 7b, black and red lines). In contrast, for the competition-only model, heterogeneous dispersal increased nestedness relative to homogeneous dispersal rates between local networks (Figure 7b, green and blue lines). However, modularity patterns were less clear. Across all interaction types and dispersal scenarios, there were no consistent differences in modularity for both meta-networks and the local networks (Figure 8).

## 3. Discussion

Understanding how mutualistic interactions and dispersal shape ecological meta-networks is crucial for advancing both theory and practice in biodiversity conservation and ecosystem resilience [7,17,18]. Our study provides new insights into how these processes jointly influence network stability, structure, and species abundance patterns, emphasising the role of dispersal heterogeneity across space and species. By integrating mutualism, competition, and dispersal dynamics, we demonstrate how local and regional processes interact to govern properties in meta-networks.

### 3.1. Mutualistic Interactions Enhance Meta-Network Stability and Structure

Our findings confirm the critical role of mutualism in stabilising ecological networks. Meta-networks incorporating mutualistic interactions were consistently more stable, more abundant, and more nested, and exhibited lower unevenness in species abundances than those with purely competitive interactions (Figure 2, Figure 3, Figure 4 and Figure 5 and Figure 7). This suggests that mutualism facilitates coexistence and resilience across spatially structured communities, a result that aligns with recent empirical and theoretical advances [17,21,43]. Interestingly, our results contrast earlier theoretical predictions that mutualism can be destabilising [19], likely because they focused on local networks without considering spatial dynamics. In our modelling framework, mutualistic interactions provide stabilising feedbacks by promoting species coexistence through dispersal-mediated processes, such as source–sink dynamics [44]. This highlights the importance of incorporating spatial structure and movement into analyses of ecological stability.

Furthermore, mutualism enhanced total species abundance and promoted a more equitable distribution of species abundances [45]. This indicates that mutualistic interactions not only boost productivity but also foster evenness, potentially reducing dominance by any single species. In our meta-network models, total species abundance was consistently higher in systems incorporating mutualism compared to those with only competitive interactions (Figure 4), due to the positive effects of mutualism on spatially coupled species, which enhances their persistence and abundance and supports biodiversity and community assemblages [18]. Mutualistic networks also exhibited higher levels of nestedness, reflecting a nested structure where generalist species facilitate interactions for specialist species, leading to a stable and resilient network [46]. However, modularity did not show a consistent pattern, possibly reflecting challenges in detecting modules in networks with low connectance and small size [47]. This may represent a limitation in our analyses, as we used only 50 species per local network, with a connectance of 0.2, which likely reduced the resolution necessary to detect modular structures.

### 3.2. Dispersal Stabilises Networks Through Complementary Mechanisms

Dispersal stabilises both local and meta-networks through two primary mechanisms: (i) the negative feedback effect and (ii) the group-distance effect. The negative feedback effect occurs when increasing dispersal rates make the diagonal elements of the Jacobian matrix more negative (Supplementary Figure S2), thus enhancing the intraspecific feedback and self-regulation strength of species [24,25]. On the other hand, the group-distance effect arises when dispersal causes the eigenvalue distribution of the Jacobian matrix in the complex plane to split into two distinct groups. Specifically, when sorted in increasing order, the first group comprises the first Sn−S eigenvalues, and the second group contains the last S eigenvalues, where S=M+N is the total number of species and n is the number of local networks, thus creating a distance between the two groups (Supplementary Figure S3).

For local networks, only the negative feedback effect contributes to increasing stability, resulting in a roughly linear relationship with dispersal (Figure 3b). However, in the meta-networks, both mechanisms are at play. Initially, at low dispersal rates, the group distance is negligible or near zero (Supplementary Figure S4, measured as the absolute difference between the last eigenvalue of the first group, the $\left(Sn-S\right)th$ eigenvalue, and the first eigenvalue of the second group, that is, the $\left(Sn-S+1\right)th$ eigenvalue), enabling the negative feedback effect to dominate and increase stability. As dispersal rates reach intermediate levels, the group distance begins to expand gradually, leading to a peak in stability as source–sink dynamics emerge across the meta-network. Beyond this, at high dispersal rates, the group distance becomes substantial, causing stability to plateau (Supplementary Figure S5). In this phase, increasing dispersal no longer boosts stability (Figure 3a), but merely widens the gap between the two eigenvalue groups (Supplementary Figure S3), with the group distance and dispersal rate exhibiting a strong positive correlation (r=0.99, Supplementary Figure S4). At high levels of dispersal rates, the spatial synchrony effect becomes prominent, effectively homogenising the meta-network and causing it to behave like a single interconnected system [48,49].

### 3.3. Dispersal Heterogeneity Between Local Networks Drives Species Abundance, Evenness, and Similarity Patterns

The effect of dispersal on network structures depends greatly on the dispersal heterogeneities between local networks. In both the competition-only and competition–mutualism model scenarios, heterogeneous dispersal rates between local networks reduced total species abundance in the meta-network (Figure 4). This likely resulted from increased variation in dispersal rates (Supplementary Figure S6, all solid lines). In contrast, homogeneous dispersal rate between local networks increased total abundance, suggesting that equal immigration into each local network sustains species interactions and promotes higher overall abundances in the meta-network [22,23].

Additionally, heterogeneous dispersal rates between local networks increased the unevenness in species abundance within local networks (Figure 5). In the meta-network, this unevenness pattern was observed in the competition-only model but not in the competition–mutualism model, likely due to the stabilising effect of mutualism (Figure 5a, black and red lines). Also, heterogeneous dispersal rates between local networks reduced spatial similarity in species abundance among local networks (Figure 6b, solid lines) and decreased temporal similarity in meta-network species abundance after dispersal (Figure 6a, solid lines). In contrast, homogeneous dispersal rates between local networks enhanced abundance and spatial and temporal similarity, indicating that equal dispersal rates promote synchrony and resilience across the meta-network. The overall pattern, where heterogeneity in dispersal between local networks reduces total abundance and spatial and temporal similarity, and increases unevenness, can be attributed to increased variation in dispersal rates (Supplementary Figure S6, solid lines).

In comparison, dispersal heterogeneity across species exhibited a weaker or negligible effect on the networks. For example, under homogeneous dispersal between local networks, two contrasting patterns emerged, depending on dispersal variability across species: (i) Heterogeneous dispersal rates across species increased total abundance (Figure 4, green and black dashed lines), suggesting that differences in dispersal ability among species can amplify species interactions across networks, thereby boosting meta-network abundance. (ii) Homogeneous dispersal rates across species had a minimal impact on total abundance (Figure 4, blue and red dashed lines; blue dashed lines for competition-only), suggesting that species abundance may become synchronised across networks when all species disperse at the same rate and with equal connectivity among local networks [23].

Despite these contrasting patterns, both heterogeneous and homogeneous dispersal rates across species converged to similar outcomes in models with mutualism (Figure 4, black and red dashed lines) and in models without mutualism (Figure 4, blue and green dashed lines). This indicates that dispersal variability across species plays a limited role; what matters more is the variability in dispersal between local networks.

Similarly, for patterns such as unevenness, spatial and temporal similarity, and nestedness, dispersal heterogeneity across species had little effect, as these patterns were mostly driven by dispersal heterogeneity between local networks or by mutualism. A minor effect was noted, that variability in dispersal across species slightly reduced nestedness in local networks, regardless of whether species dispersed at different (Figure 7, green dashed line) or similar rates (Figure 7, blue dashed line).

In summary, patterns in species abundance, unevenness, and spatial and temporal similarities were mainly driven by dispersal heterogeneity between local networks, while dispersal heterogeneity across species played a secondary role.

These findings offer insights for applied ecology, especially in the conservation of pollinators, plants, and the design of ecological networks [50,51]. For example, the limited effects of dispersal heterogeneity between local networks highlight the value of maintaining well-connected and balanced habitats. Hence, promoting more uniform dispersal pathways, such as through ecological corridors [52], may help sustain biodiversity and community structure in fragmented landscapes [53]. Also, understanding how mutualistic interactions respond to spatial structures can inform conservation strategies that are aimed at protecting plant–pollinator systems, which are vital for ecosystem services and food security [54].

## 4. Materials and Methods

### 4.1. Meta-Network Model

Consider a meta-network comprised of n interlinked local networks, each hosting interacting populations of M number of plant species and N number of animal species. The abundance of plant species i, in local network k, is denoted by $P_{ik}$ (for i=1,…,M and k=1,…, n), and the abundance of animal species j in local network k is denoted by $A_{jk}$ (for j=1,…,N). To describe the population dynamics within and between the local networks, we model intra-guild competition (which is among plants or among animals within a local network), inter-guild mutualistic interactions (between plants and animal species within a local network), and dispersal, which is the movement of individuals between local networks.

The mutualistic interactions are modelled using a Holling type II functional response to account for saturation in the benefits a species obtains from its partners [55]; a similar approach has been used for implementing multispecies mutualistic interactions [15,42]. Dispersal is modelled with a linear term representing emigration and immigration between local networks. Hence, the model is defined by the following system of differential equations:

(1) $$\frac{dP_{ik}}{dt}=\left[r_{ik}\;\left(1-\sum_{j=1}^{M}\alpha_{ijk}^{\left(P\right)}P_{jk}\right)+\frac{\sum_{j=1}^{N}a_{ij}\beta_{ijk}^{\left(P\right)}A_{jk}}{1+h\sum_{j=1}^{N}a_{ijk}A_{jk}}\right]P_{ik}-\sum_{\begin{array}{c} l=1\\ l\neq k \end{array}}^{n-1}{d_{ilk}^{\left(P\right)}P}_{ik}+\sum_{\begin{array}{c} l=1\\ l\neq k \end{array}}^{n-1}{d_{ikl}^{\left(P\right)}P}_{il}$$

(2) $$\frac{dA_{jk}}{dt}=\left[r_{jk}\;\left(1-\sum_{i=1}^{N}\alpha_{jik}^{\left(A\right)}A_{ik}\right)+\frac{\sum_{i=1}^{M}a_{ij}\beta_{jik}^{\left(A\right)}P_{ik}}{1+h\sum_{i=1}^{M}a_{ijk}P_{ik}}\right]A_{jk}-\sum_{\begin{array}{c} l=1\\ l\neq k \end{array}}^{n-1}{d_{jlk}^{\left(A\right)}A}_{jk}+\sum_{\begin{array}{c} l=1\\ l\neq k \end{array}}^{n-1}{d_{jkl}^{\left(A\right)}A}_{jl}$$

where $\left(P\right)$ and $\left(A\right)$ are superscripts for plants and animals. $dP_{ik}/dt$ is the rate of change in the abundance of plant species i in local network k; $dA_{jk}/dt$ is the rate of change in the abundance of animal species j in local network k; $r_{ik}$ and $r_{jk}$ are the growth rates of plant i and animal j, respectively, in local network k; $\alpha_{ijk}^{\left(P\right)}\;$ is the competition strength between plant species i and j in local network k, where $\alpha_{iik}^{\left(P\right)}$ is the self-regulation of plant species i in local network k; $\alpha_{jik}^{\left(A\right)}\;$ is the competition strength between animal species j and i in local network k, where $\alpha_{jjk}^{\left(A\right)}$ is the self-regulation of animal species i in local network k; $a_{ij}\;$ is the binary interaction matrix between plant species i and animal j; $\beta_{ijk}^{\left(P\right)}\;$ is the benefit obtained by plant species i from animal species j in local network k; $\beta_{jik}^{\left(A\right)}\;$ is the benefit obtained by animal species j from plant species i in local network k; h is the handling time and assumed to be the same for all species. The last two terms in the equations are the emigration out of and the immigration into local network k, respectively, where $d_{ilk}^{\left(P\right)}$ and $d_{jlk}^{\left(A\right)}$ are the dispersal rates of plant species i and of animal species j moving from local network k to local networks l; $d_{ikl}^{\left(P\right)}$ and $d_{jkl}^{\left(A\right)}$ are the dispersal rates of plant species i and of animal species j moving from local networks l to local network k. Note, when the mutualism benefits are assigned to zero, the model corresponds to the competition-only meta-network model.

### 4.2. Dispersal Heterogeneity Between Local Networks and Across Species

Different species can have different dispersal rates in a community. To incorporate this dispersal heterogeneity, we considered variation in dispersal rates in two distinct ways: (1) between local networks, and (2) across species within a local network.

For each species i, we define an n×n dispersal matrix D, where the diagonal element $d_{ikk}$ represents the emigration rate of species i out of local network k, and the off-diagonal elements $d_{ikl}$ represent the immigration of species i from local network l to local network k. Assuming all local networks are connected, suppose there are three local networks; n=3, then $d_{i11}$ is the rate of species i emigrating out of local network 1, $d_{i21}$ and $d_{i31}$ are, respectively, the dispersal rates of species i moving from local network 1 to local network 2 and from local network 1 to local network 3. Thus, the rates are partitioned such that $d_{i11}=d_{i21}+d_{i31}$. Heterogeneous dispersal rates between local networks are considered here when the emigration rate $d_{i11}$ is randomly partitioned into $d_{i21}$ and $d_{i31}$ such that $d_{i21}\neq d_{i31}$, while homogeneous dispersal rates between local networks are considered when the emigration rate is evenly distributed such that $d_{i21}=d_{i31}=d_{i11}/(n-1)$.

We also consider variation in dispersal rates across species, independent of their location in the meta-network. That is, different species may have different dispersal capabilities. For example, invasive species might have a higher dispersal rate than other, native, species due to invasive traits such as higher mobility [56]. To model this, the emigration rate $d_{ikk}$ for each species i in local network k is drawn from a normal distribution with mean $\mu_{d}$ and standard deviation $\sigma_{d}$. A large value of $\sigma_{d}$ represents heterogeneous dispersal rates across species, while a small value of $\sigma_{d}$ represents homogeneous dispersal rates across species in the local network.

We then considered these four dispersal scenarios, including heterogeneous or homogeneous dispersal between local networks, and heterogeneous or homogeneous dispersal across species in a local network, in the competition–mutualism or competition-only interaction structures. These eight model scenarios are summarised in Figure 1b.

### 4.3. Simulation and Parameterisation

We solved the system of differential Equations (1) and (2) using the *lsoda* solver implemented in the *desolve* 1.30 package in R (version 4.0.1). The parameters were assigned from uniform $U\left(x,y\right)$, normal $N\left(\mu ,\sigma \right)$, and log-normal $LN\left(\mu ,\sigma \right)$ distributions, where *x* and *y* are the minimum and maximum elements of the uniform distribution, respectively; μ and σ are the mean and standard deviation of the normal and log-normal distributions. Parameters and their respective assigned values are described in Table 1.

We simulated 50 species (comprising 30 plant and 20 animal species) in each of 10 local networks. For each of the eight model scenarios (arising from combinations of interaction type and dispersal heterogeneity), we evaluated 30 values of the mean dispersal rate $\mu_{d}$, ranging from $e^{-4}\;to\;e^{3}$, in logarithmic steps of size $e^{0.25}$. Each simulation was run over 50 timesteps, sufficient for the system to attain equilibrium. After reaching equilibrium, network structures and other metrics were computed for analysis.

### 4.4. Network Structures and Metrics

#### 4.4.1. Stability Measured by the Leading Eigenvalue $\lambda_{m}$

Stability was assessed by computing the leading eigenvalue of the Jacobian matrix obtained by linearising the system of equations (1) and (2) at equilibrium abundance (see Supplementary Text S1 for details). For the Meta-network, we considered the real part of the leading eigenvalue of the full Jacobian matrix J (see Supplementary Equation (S2)), while, for each of the local networks, we considered the real part of the leading eigenvalue of the local Jacobian submatrix $J_{kk}$ (see Supplementary Equation (S3)). A system is considered more locally stable when the real part of the leading eigenvalue is more negative.

The diagonal elements of $J_{kk}$ (see Supplementary Equation (S4)) play an important role in determining local stability. For example, on the plant side, they are given by

(3) $${\left.\frac{\partial {\dot{P}}_{ik}}{\partial P_{ik}}\right|}_{\vec{X}}=-r_{ik}\alpha_{iik}^{\left(P\right)}P_{ik}-\frac{1}{P_{ik}}\sum_{\begin{array}{c} l=1\\ l\neq k \end{array}}^{n-1}d_{ikl}^{\left(P\right)}P_{il}$$

where the first term on the right-hand side is the intrinsic growth rate $r_{ik}$ and the self-regulation $\alpha_{iik}^{\left(P\right)}$ of plant species i in local network k, while the second term represents the sum of all immigration from other local networks (indexed by l) into local network k for plant species i. Therefore, as the dispersal rate increases, the diagonal elements become more negative. This phenomenon is known as the *negative feedback effect* of dispersal (e.g., [24]) and has a stabilising effect. A foreseeable effect, according to the circular law [57], is that such increases in dispersal rate will shift the centre of the eigenvalues of the meta-network towards left in the complex plane, thus making it more stable when other factors are not considered (Supplementary Figure S3).

#### 4.4.2. Total Abundance

The total abundance was computed as the sum of the equilibrium abundances of all species across all local networks, given by

(4) $$\sum_{k=1}^{n}\sum_{i=1}^{S}X_{ik}^{*}$$

where $X_{ik}^{*}$ is the equilibrium abundance of species i in local network k, while S is the total number of all the species (S=M+N) and n is the number of local networks.

#### 4.4.3. Unevenness Measured by Gini Coefficient

To determine the level of unevenness in the abundance of species in a network, we used the Gini coefficient [58]. This metric captures how evenly or unevenly abundance is distributed among species in a network. A perfectly even network, where all species have equal abundance, yields a Gini coefficient of 0. Conversely, a value close to 1 indicates high unevenness, where a few species dominate in abundance while others are rare. When the equilibrium abundances of the species in a network are ranked in an increasing order $(X_{i}^{*}<X_{i+1}^{*}$), the Gini coefficient is given by

(5) $$G=\frac{1}{S}\left(S+1-2\frac{\sum_{i=1}^{S}\left(S+1-i\right)X_{i}^{*}}{\sum_{i=i}^{S}X_{i}^{*}}\right)$$

where $X_{i}^{*}$ is the equilibrium abundance of species i, and S is the number of all species in the network. We measured the unevenness in the abundance of species without dispersal ($\mu_{d}=0$, $\sigma_{d}=0$) and with dispersal ($\mu_{d}\neq 0$, $\sigma_{d}\neq 0$) over the meta-network scale and the local network scale. At each dispersal mean value, $\mu_{d}$ in the simulation, for the meta-network, we computed the Gini coefficient using the equilibrium abundances of all species across all local networks. For the local networks, we computed the Gini coefficient for each local network and then averaged the values across all n local networks.

#### 4.4.4. Compositional Similarity Measured by Morisita–Horn Index

To determine the compositional similarity between networks, we used the Morisita–Horn similarity index [59,60], a quantitative measure for assessing species composition between networks. Using this index, we compared the relative abundance at the equilibrium of species in a network either spatially across local networks or temporarily before and after implementing dispersal in a meta-network. Spatial and temporal are used metaphorically.

For the spatial similarity across local networks, the Morisita–Horn similarity index measures the closeness of the relative abundance at the equilibrium of species in different local networks, by comparing the relative abundance at equilibrium of each species in each local network [30], given by

(6) $$C_{2n}=\frac{2\sum_{i=1}^{S}\sum_{\begin{array}{c} j<k\\ j=1\\ k=j+1 \end{array}}^{n}p_{ij}p_{ik}}{\left(n-1\right)\sum_{i=1}^{S}\sum_{j=1}^{n}p_{ij}^{2}}$$

where S and n are the numbers of species and local networks, respectively, $p_{ij}$ is the relative abundance of species i in network j, and $p_{ik}$ is the relative abundance of species i in local network k. Note, $C_{2n}=0$ represents the case when the local networks share no species, while $C_{2n}=1$ is when the local networks are compositionally identical.

For the temporal similarity between the meta-networks, we compared the closeness of the relative abundance at the equilibrium of species in the meta-network before implementing dispersal (that is, when the dispersal rate is zero) and after implementing dispersal. The index is given by

(7) $$C_{22}=\frac{2\sum_{i=1}^{S}q_{ib}q_{ia}}{\sum_{i=1}^{S}q_{ib}^{2}+q_{ia}^{2}}$$

where S is the number of species, $q_{ib}$ is the relative abundance of species i in the meta-network before implementing dispersal, and $q_{ia}$ is the relative abundance of species i in the meta-network after implementing dispersal. Note, $C_{22}=0$ represents when the meta-networks share no species, while $C_{22}=1$ represents when the meta-networks are identical.

#### 4.4.5. Nestedness and Modularity

To determine the level of nestedness and modularity, we used a weighted interaction matrix, representing a bipartite network. The nestedness of the bipartite network is the degree to which specialist species are subsets of species that interact with generalist species in the network [61]. On the other hand, the modularity of a bipartite network is the degree to which the interactions in a network cluster into modules of tightly interacting species than is expected by chance. That is, for a modular network, interactions occur within modules more frequently than between modules [47].

For each of the local networks, the weighted interaction matrix is obtained by multiplying the binary interaction matrix by the equilibrium abundance of the species. This is given by $a_{ij}P_{ik}^{*}A_{jk}^{*}$, where $a_{ij}$ is the binary interaction matrix, $P_{ik}^{*}$ is the equilibrium abundance of plant species i in local network k, and $A_{jk}^{*}$ is the equilibrium abundance of animal species j in local network k. For the meta-network, the weighted interaction matrix is obtained by multiplying the binary interaction matrix by the total sum of the equilibrium abundance across all local networks, given by $\sum_{k=1}^{n}a_{ij}P_{ik}^{*}A_{jk}^{*}$. This allows for a useful representation of the weighted interactions between species, incorporating the effect of equilibrium abundances.

Both nestedness and modularity were implemented using R package *bipartite* 2.11 [62]. Specifically, nestedness was computed using ‘weighted NODF’ (weighted nestedness based on overlap and decreasing fill; [63]), and modularity was computed using ‘LPA_wb_plus’ (label propagation and multi-step agglomeration that maximise modularity in weighted networks; [64]).

## 5. Conclusions

Network approaches have been useful in uncovering structural patterns in species interactions within complex multispecies systems [27,61]. Integrating spatial and temporal dynamics into these frameworks offers valuable insights for species conservation and ecosystem restoration [11]. In this research, we have demonstrated that incorporating mutualism in models of meta-networks is crucial for network stability. Mutualistic interactions not only enhance the stability of meta-networks but also increase total species abundance, reduce unevenness in species abundance, and promote higher levels of nestedness in both local and meta-networks. Also, dispersal emerged as a key stabilising factor for the meta-network, operating through both the negative feedback effect and the group-distance effect. In addition, we have shown that dispersal heterogeneity between local networks drives the changes in total abundance, unevenness, and spatial and temporal similarity than dispersal heterogeneity across species in the meta-network and the local network. That is, dispersal heterogeneity between the local networks reduces total abundance, increases unevenness, and decreases compositional similarity in the meta-networks and local networks.

The novelty of our work lies in disentangling the effects of mutualism and two forms of dispersal heterogeneity, which are between local networks and across species, on multiple structural and stability metrics in plant–pollinator meta-networks. Hence, these findings underscore the critical role of understanding dispersal rates both between local networks and across species, in shaping the structure and stability of ecological networks. Such insights are particularly relevant for developing effective strategies for habitat management and conservation.

### Limitations and Future Directions

Our model incorporates several simplifying assumptions that, while necessary for analytical tractability, limit its ecological realism and warrant further investigation. We assumed that all local networks are fully connected, thereby ignoring potential spatial barriers, the cost of dispersal, and landscape fragmentation, which are likely to influence real-world dynamics [65]. Additionally, the model neglects habitat heterogeneity, environmental fluctuations, and stochasticity, which are integral to natural systems and could modify both the strength and direction of the observed effects. Furthermore, the model focuses mainly on symmetric mutualistic interactions between plants and pollinators, neglecting other interaction types. While these simplifications enabled us to isolate key mechanisms, they also reduced the model’s direct applicability to complex natural systems. Further work should relax these assumptions, incorporate empirical data, and further test how environmental variability interacts with dispersal and mutualism to shape ecological networks. In addition, experimental studies and field data are essential to validate our predictions, especially regarding the stabilising roles of mutualistic interactions and dispersal heterogeneity.

## Figures and Tables

**Table 1:** Description of the model parameters, their meaning, assigned values, and units.

Parameters	Meaning	Assigned Values	Units
$a_{ij}$	Binary interaction from animal species j to plant species i	1—presence or 0—absence of an interaction, assigned with a connectance C=0.2	–
$P_{ik}$	Population abundance of plant species i in local network k	$U\left(0,1\right)$	–
$A_{jk}$	Population abundance of animal species j in local network k	$U\left(0,1\right)$	–
$r_{ik}$	Intrinsic growth rate of species i in local network k	$LN\left(1,0.1\right)$	${time}^{-1}$
$\alpha_{ijk}$	Competition strength from species j to i in local network k	$\left|N\left(\mu_{1},\sigma_{1}\right)\right|=\left|N\left(0,0.05\right)\right|$	–
$\beta_{ijk}$	Mutualism benefit strength obtained by species i from species j in local network k	$\left|N\left(\mu_{2},\sigma_{2}\right)\right|=\left|N\left(0,0.05\right)\right|$	–
$\alpha_{iik}$	Self-regulation of species i in local network k	−1	–
h	Handling time	0.1	time
$d_{ikk}$	Dispersal rate of species i out of local network k	$N\left(\mu_{d},\sigma_{d}\right)$	${time}^{-1}$
$\mu_{d}$	Mean of dispersal rate	$\left[0,e^{-4},\ldots ,e^{3}\right]$ $\mathrm{step}\;\mathrm{size}=e^{0.25}$	–
$\sigma_{d}$	Standard deviation of dispersal rate across species	0.01—for homogeneous, and 0.5—for heterogeneous	–
n	Number of local networks	10	–

## Data Availability

The R scripts can be found in https://github.com/AssumptaOnye/R-script/blob/main/Rscript_mutualistic_metanetwork.R (accessed on 26 May 2025).

## Supplementary Materials

The following supporting information can be downloaded at https://www.mdpi.com/article/10.3390/plants14142127/s1. 1. Supplementary Text S1: Stability analysis; 2. Supplementary Figure S1: Comparison of the different interaction types using the general Lotka-Volterra model; Supplementary Figure S2: The mean of the diagonal elements of the Jacobian matrix: illustrating the negative feedback effect of dispersal rate; Supplementary Figure S3: The eigenvalue distribution of the Jacobian matrix of the meta-networks: illustrating the group-distance effect of dispersal rate; Supplementary Figure S4: The relationship between the group distance and the dispersal rate of the meta-networks; Supplementary Figure S5: The relationship between the leading eigenvalues and the group distance in the meta-networks; Supplementary Figure S6: The standard deviation of the diagonal elements of the Jacobian matrix.
