# Supplementary material for: Mutualism and Dispersal Heterogeneity Shape Stability, Biodiversity, and Structure of Theoretical Plant–Pollinator Meta-Networks
Source: Plants (Basel). 2025 Jul 10;14(14):2127. doi: 10.3390/plants14142127 (PMC12300112; doi:10.3390/plants14142127)
Supplement: Supplementary file 1 [file plants-14-02127-s001.zip › plants-3703867-supplementary.pdf]

Supplementary Material

Contents

1. Supplementary Text S1. Stability Analysis
2. Supplementary Figure S1. Comparison of the different interaction types using the general Lotka-Volterra model.
3. Supplementary Figure S2. The mean of the diagonal elements of the Jacobian matrix: illustrating the negative feedback effect of dispersal rate.
4. Supplementary Figure S3. The eigenvalue distribution of the Jacobian matrix of the meta-networks: illustrating the group-distance effect of dispersal rate.
5. Supplementary Figure S4. The relationship between the group-distance and the dispersal rate of the meta-networks.
6. Supplementary Figure S5. The relationship between the leading eigenvalues and the group-distance in the meta-networks.
7. Supplementary Figure S6. The standard deviation of the diagonal elements of the Jacobian matrix.

Supplementary Text S1

Stability Analysis

The stability of both the meta-network and of the local networks was assessed using the leading eigenvalue of the Jacobian matrix. Suppose the equilibrium abundance exists, the Jacobian matrix is obtained by linearising the system of equations (1) and (2) (in the main text) at the equilibrium abundance

$$\vec{X} = (P_{11}^*, \dots, P_{M1}^*, A_{11}^*, \dots, A_{N1}^*, P_{12}^*, \dots, P_{M2}^*, A_{12}^*, \dots, A_{N2}^*, \dots, P_{1n}^*, \dots, P_{Mn}^*, A_{1n}^*, \dots, A_{Nn}^*).$$

Then, the dynamical behaviour of small perturbations from the equilibrium abundance $\vec{X}$ is given by:

$$\frac{\boldsymbol{dx}}{\boldsymbol{dt}} = \boldsymbol{Jx}\,, \tag{S1}$$

where **x** is the displacement from equilibrium and **J** is the Jacobian matrix of the meta-network described by:

$$\boldsymbol{J} = \begin{bmatrix} \mathrm{J}_{11} & \mathrm{J}_{12} & \cdots & \mathrm{J}_{1n} \\ \mathrm{J}_{21} & \mathrm{J}_{22} & \cdots & \mathrm{J}_{2n} \\ \vdots & \vdots & \ddots & \vdots \\ \mathrm{J}_{n1} & \mathrm{J}_{n2} & \cdots & \mathrm{J}_{nn} \end{bmatrix} \tag{S2}$$

The elements of **J** are the $(M + N) \times (M + N)$, submatrices J_{kl} $(k, l = 1, \dots, n)$, where J_{kk} is the diagonal submatrix and represents the Jacobian matrix of the kth local network, which captures the interactions within the local network:

$$
J_{kk} = \begin{bmatrix} j_{PP} & j_{PA} \\ j_{AP} & j_{AA} \end{bmatrix} = \begin{bmatrix} \frac{\partial \dot{P}_{1k}}{\partial P_{1k}} & \cdots & \frac{\partial \dot{P}_{1k}}{\partial P_{Mk}} & \frac{\partial \dot{P}_{1k}}{\partial A_{1k}} & \cdots & \frac{\partial \dot{P}_{1k}}{\partial A_{Nk}} \\ \vdots & \ddots & \vdots & \vdots & \ddots & \vdots \\ \frac{\partial \dot{P}_{Mk}}{\partial P_{1k}} & \cdots & \frac{\partial \dot{P}_{Mk}}{\partial P_{Mk}} & \frac{\partial \dot{P}_{Mk}}{\partial A_{1k}} & \cdots & \frac{\partial \dot{P}_{Mk}}{\partial A_{Nk}} \\ \frac{\partial \dot{A}_{1k}}{\partial P_{1k}} & \cdots & \frac{\partial \dot{A}_{1k}}{\partial P_{Mk}} & \frac{\partial \dot{A}_{1k}}{\partial A_{1k}} & \cdots & \frac{\partial \dot{A}_{1k}}{\partial A_{Nk}} \\ \vdots & \ddots & \vdots & \vdots & \ddots & \vdots \\ \frac{\partial \dot{A}_{Nk}}{\partial P_{1k}} & \cdots & \frac{\partial \dot{A}_{Nk}}{\partial P_{Mk}} & \frac{\partial \dot{A}_{Nk}}{\partial A_{1k}} & \cdots & \frac{\partial \dot{A}_{Nk}}{\partial A_{Nk}} \end{bmatrix} \tag{S3}
$$

In general, elements of J_{kk} describe the partial derivatives of the rate of abundance change of each species with respect to the abundance of the species in local network k, and can be obtained as follows:

$$
J_{kk}^{\text{elements}} = \begin{cases}
\left.\frac{\partial \dot{P}_{ik}}{\partial P_{ik}}\right|_{\vec{X}} = -r_{ik}\alpha_{iik}^{(P)}P_{ik} - \frac{1}{P_{ik}}\sum_{\substack{l=1 \\ l\neq k}}^{n-1} d_{ikl}^{(P)}P_{il}\,, \quad i = 1, \ldots, M, \quad \text{for the diagonal of } j_{PP} \\
\left.\frac{\partial \dot{A}_{jk}}{\partial A_{jk}}\right|_{\vec{X}} = -r_{jk}\alpha_{jjk}^{(A)}A_{jk} - \frac{1}{A_{jk}}\sum_{\substack{l=1 \\ l\neq k}}^{n-1} d_{jkl}^{(A)}A_{jl}\,, \quad j = 1, \ldots, N, \quad \text{for the diagonal of } j_{AA} \\
\left.\frac{\partial \dot{P}_{ik}}{\partial P_{jk}}\right|_{\vec{X}} = -r_{ik}\alpha_{ijk}^{(P)}P_{ik}\,, \quad i, j = 1, \ldots, M, \quad \text{for the offdiagonal of } j_{PP} \\
\left.\frac{\partial \dot{A}_{jk}}{\partial A_{ik}}\right|_{\vec{X}} = -r_{jk}\alpha_{ijk}^{(A)}A_{jk}\,, \quad j, i = 1, \ldots, N, \quad \text{for the offdiagonal of } j_{AA} \\
\left.\frac{\partial \dot{P}_{ik}}{\partial A_{jk}}\right|_{\vec{X}} = P_{ik}\left(\frac{\left(1 + h\sum_{j=1}^{N} a_{ij}A_{jk}\right)\left(a_{ij}\beta_{ijk}^{(P)}\right) - \left(\sum_{j=1}^{N} a_{ij}\beta_{ijk}^{(P)}A_{jk}\right)\left(h\, a_{ij}\right)}{\left(1 + h\sum_{j=1}^{N} a_{ij}A_{jk}\right)^2}\right), \\
\qquad i = 1, \ldots, M, \quad j = 1, \ldots, N, \quad \text{for } j_{PA} \\
\left.\frac{\partial \dot{A}_{jk}}{\partial P_{ik}}\right|_{\vec{X}} = A_{jk}\left(\frac{\left(1 + h\sum_{i=1}^{M} a_{ij}P_{ik}\right)\left(a_{ij}\beta_{ijk}^{(A)}\right) - \left(\sum_{i=1}^{M} a_{ij}\beta_{jik}^{(A)}P_{ik}\right)\left(h\, a_{ij}\right)}{\left(1 + h\sum_{i=1}^{M} a_{ij}P_{ik}\right)^2}\right), \\
\qquad j = 1, \ldots, N, \quad i = 1, \ldots, M, \quad \text{for } j_{AP}
\end{cases} \tag{S4}
$$

In the same way the off-diagonal submatrices J_{kl} ($k \neq l$) capture dispersal effects between local networks and are defined as:

$$J_{kl} = \begin{bmatrix} d_{PP} & d_{PA} \\ d_{AP} & d_{AA} \end{bmatrix} = \begin{bmatrix} \frac{\partial \dot{P}_{1k}}{\partial P_{1l}} & \cdots & \frac{\partial \dot{P}_{1k}}{\partial P_{Ml}} & \frac{\partial \dot{P}_{1k}}{\partial A_{1l}} & \cdots & \frac{\partial \dot{P}_{1k}}{\partial A_{Nl}} \\ \vdots & \ddots & \vdots & \vdots & \ddots & \vdots \\ \frac{\partial \dot{P}_{Mk}}{\partial P_{1l}} & \cdots & \frac{\partial \dot{P}_{Mk}}{\partial P_{Ml}} & \frac{\partial \dot{P}_{Mk}}{\partial A_{1l}} & \cdots & \frac{\partial \dot{P}_{Mk}}{\partial A_{Nl}} \\ \frac{\partial \dot{A}_{1k}}{\partial P_{1l}} & \cdots & \frac{\partial \dot{A}_{1k}}{\partial P_{Ml}} & \frac{\partial \dot{A}_{1k}}{\partial A_{1l}} & \cdots & \frac{\partial \dot{A}_{1k}}{\partial A_{Nl}} \\ \vdots & \ddots & \vdots & \vdots & \ddots & \vdots \\ \frac{\partial \dot{A}_{Nk}}{\partial P_{1l}} & \cdots & \frac{\partial \dot{A}_{Nk}}{\partial P_{Ml}} & \frac{\partial \dot{A}_{Nk}}{\partial A_{1l}} & \cdots & \frac{\partial \dot{A}_{Nk}}{\partial A_{Nl}} \end{bmatrix} \tag{S5}$$

where the elements of J_{kl}, can be obtained as follows:

$$J_{kl}^{elements} = \begin{cases} \left.\frac{\partial \dot{P}_{ik}}{\partial P_{il}}\right|_{\vec{X}} = d_{ikl}^{(P)}, \quad i = 1, \dots, M, \quad \text{for the diagonal of } d_{PP} \\ \left.\frac{\partial \dot{A}_{jk}}{\partial A_{jl}}\right|_{\vec{X}} = d_{jkl}^{(A)}, \quad j = 1, \dots, N, \quad \text{for the diagonal of } d_{AA} \\ \left.\frac{\partial \dot{P}_{ik}}{\partial P_{jl}}\right|_{\vec{X}} = 0, \quad i, j = 1, \dots, M, \quad \text{for the offdiagonal of } d_{PP} \\ \left.\frac{\partial \dot{A}_{jk}}{\partial A_{il}}\right|_{\vec{X}} = 0, \quad j, i = 1, \dots, N, \quad \text{for the offdiagonal of } d_{AA} \\ \left.\frac{\partial \dot{P}_{ik}}{\partial A_{jl}}\right|_{\vec{X}} = 0, \quad i = 1, \dots, M, \quad j = 1, \dots, N, \quad \text{for } d_{PA} \\ \left.\frac{\partial \dot{A}_{jk}}{\partial P_{il}}\right|_{\vec{X}} = 0, \quad j = 1, \dots, N, \quad i = 1, \dots, M, \quad \text{for } d_{AP} \end{cases} \tag{S6}$$

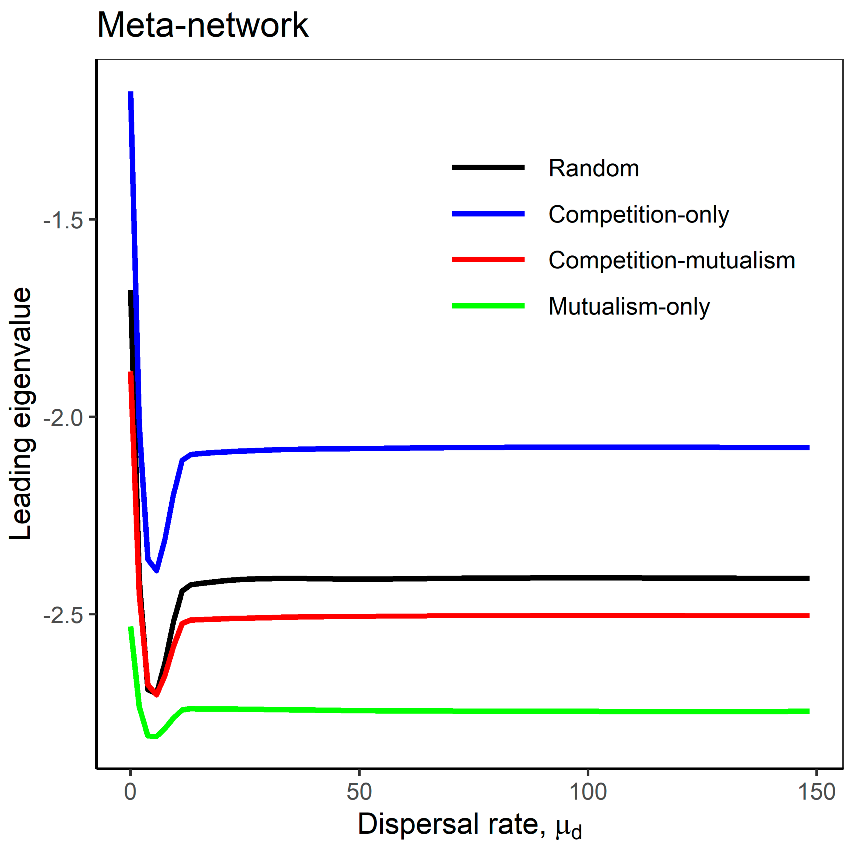

Figure S1. Comparison of the different interaction types using the general Lotka-Volterra model as implemented in Gravel *et al.* (2016). The black line represents random interactions within each local network, where interspecific interaction strengths are drawn from a normal distribution $N(0,0.1)$, as shown in Gravel *et al.* (2016). The blue line represents competition-only interactions, where interspecific interactions are drawn from $-|N(0,0.1)|$. The red line represents competition-mutualism interactions, where competition strengths and mutualism strengths are drawn from $-|N(0,0.1)|$ and $|N(0,0.1)|$, respectively. Lastly, the green line represents mutualism-only interactions, with mutualism strengths drawn from $|N(0,0.1)|$. Other fixed parameters are $S = 15; C = 0.2; m = -1;\ \mu = 0;\ \sigma = 0.1; n = 10$.

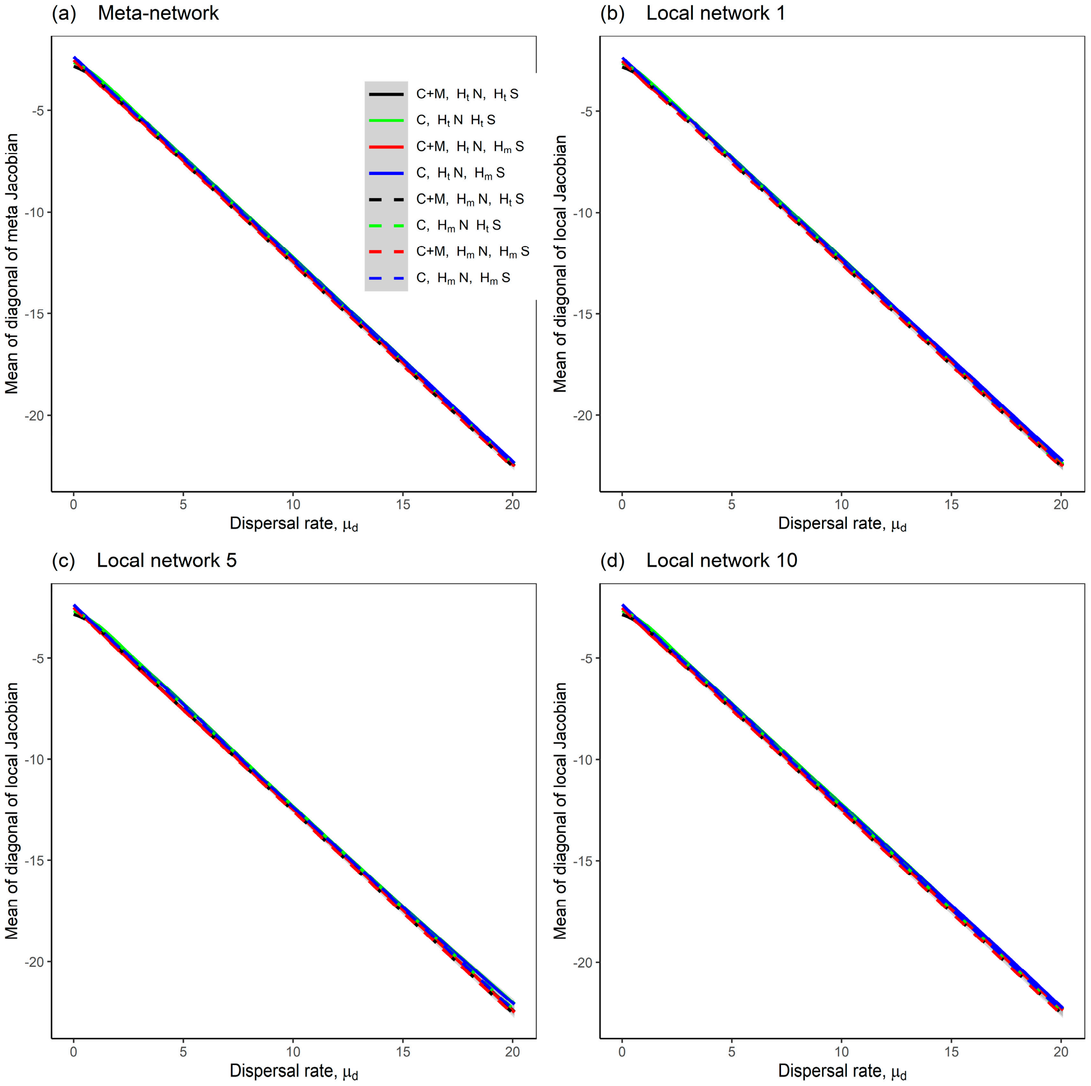

Figure S2. The mean of the diagonal elements of the Jacobian matrix: illustrating the negative feedback effect of dispersal rate. This comprises of the meta-network and three local networks as the dispersal rate, μ_d increases. The lines are showing the Loess regression where (a) is for meta-network, (b) is for local network 1, (c) is for local network 5, and (d) is for local network 10. Eight model scenarios were considered, where the solid lines are the scenarios with heterogeneous dispersal rates between the local networks, the dashed lines are the scenarios with homogeneous dispersal rates between the local networks, the black and red lines are the scenarios with competition-mutualism model, the green and blue lines are the scenarios with competition-only model, the scenarios with heterogeneous dispersal rates across species where $\sigma_d = 0.5$ are the black and green lines, while the scenarios with homogeneous dispersal rates across species where $\sigma_d = 0.01$ are the red and blue lines. A description of the scenarios is provided in Figure 1. Fixed parameters are as follows: $M = 30; N = 20; C = 0.2; m = -1$; $\mu_1 = 0$; $\sigma_1 = 0.05$; $\mu_2 = 0$; $\sigma_2 = 0.05$; $S = M + N$; $n = 10$.

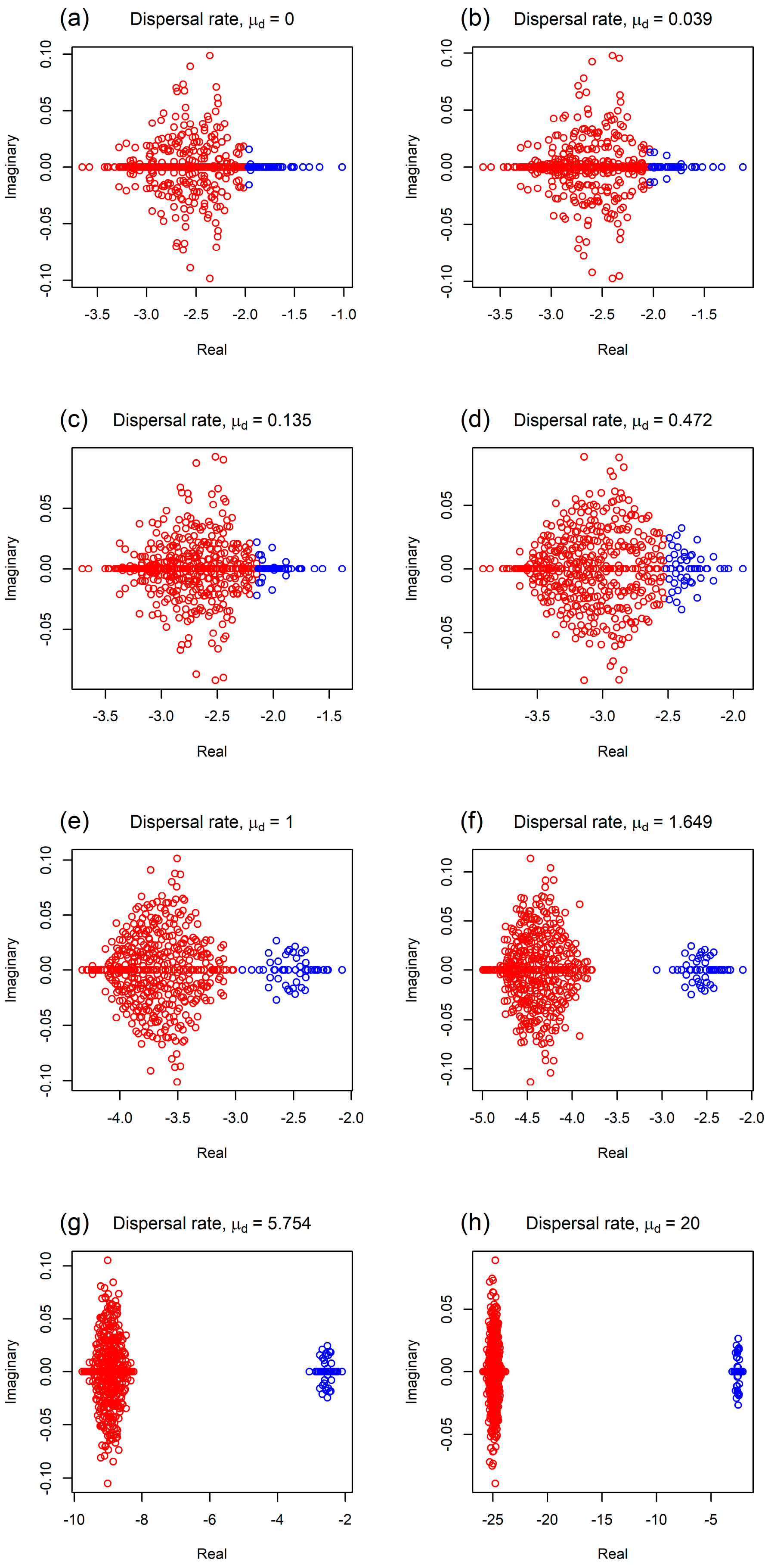
(a) Dispersal rate, μd = 0
(b) Dispersal rate, μd = 0.039
(c) Dispersal rate, μd = 0.135
(d) Dispersal rate, μd = 0.472
(e) Dispersal rate, μd = 1
(f) Dispersal rate, μd = 1.649
(g) Dispersal rate, μd = 5.754
(h) Dispersal rate, μd = 20
Imaginary
Real

Figure S3. The eigenvalue distribution of the Jacobian matrix of the meta-networks: illustrating the group-distance effect of dispersal rate. Each panel corresponds to a different dispersal rate μ_d. The distribution shows two distinct groups of eigenvalues: when sorted in decreasing order, the first $(Sn - S)$ eigenvalues are highlighted in red, while the last S eigenvalues are highlighted in blue. The model depicted corresponds to the scenario with competition-mutualism, homogeneous dispersal rate between local networks, and homogeneous dispersal rate across species $(C + M, H_m N, H_m S)$. As dispersal rate increases, the distance between these two groups also increases.

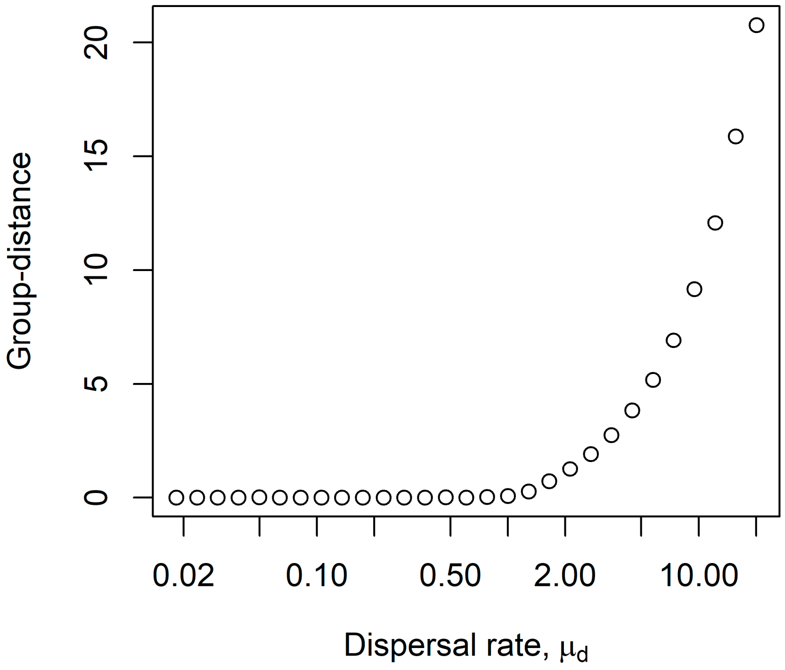

Figure S4. The relationship between the group-distance and the dispersal rate of the meta-networks. The Spearman's correlation coefficient is 0.99, indicating a strong positive relationship. When the eigenvalues are sorted in increasing order, the group-distance is measured as the absolute difference between the last eigenvalue of the first group (the $(Sn - S)'$th eigenvalue) and the first eigenvalue of the second group that is (the $(Sn - S + 1)'$th eigenvalue).

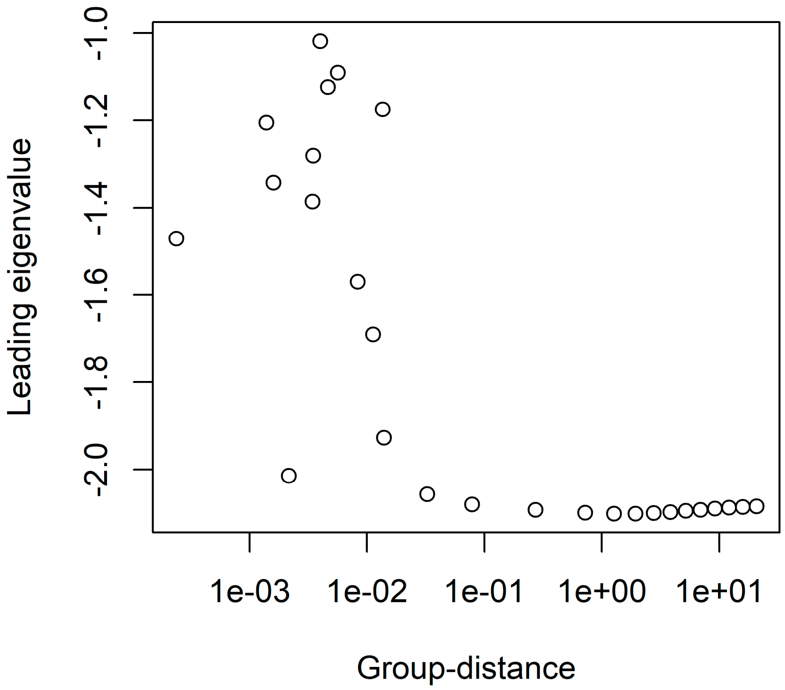

Figure S5. The relationship between the leading eigenvalues and the group-distance in the meta-networks. When the eigenvalues are sorting in increasing order, the group-distance is measured as the absolute difference between the last eigenvalue of the first group ($(Sn - S)'$th eigenvalue) and the first eigenvalue of the second group that is ($(Sn - S + 1)'$th eigenvalue).

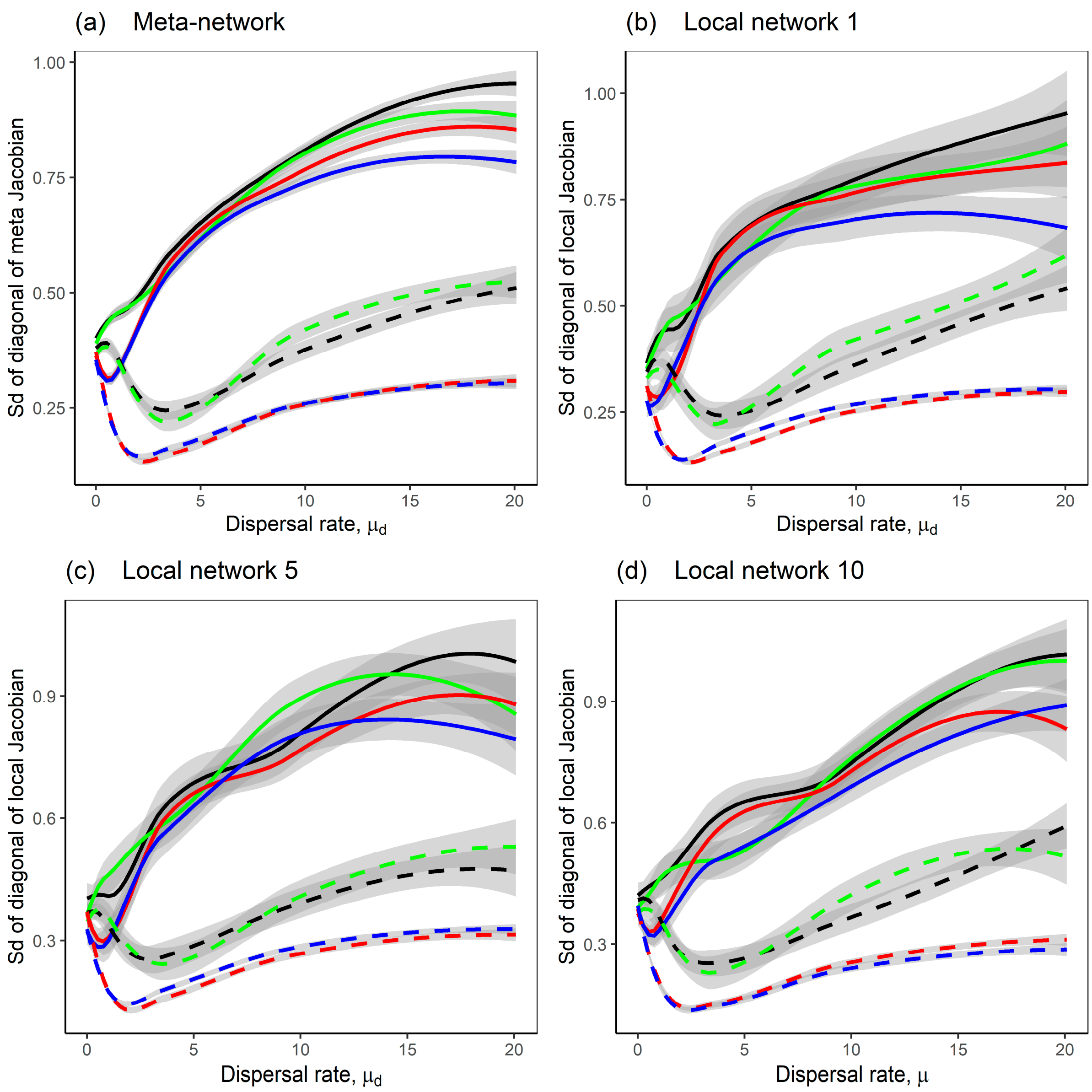

Figure S6. The standard deviation of the diagonal elements of the Jacobian matrix of the meta-network and three local networks as the dispersal rate, μ_d increases. The lines are showing the Loess regression where (a) is for meta-network, (b) is for local network 1, (c) is for local network 5, and (d) is for local network 10. Eight model scenarios were considered, where the solid lines are the scenarios with heterogeneous dispersal rates between the local networks, the dashed lines are the scenarios with homogeneous dispersal rates between the local networks, the black and red lines are the scenarios with competition-mutualism model, the green and blue lines are the scenarios with competition-only model, the scenarios with heterogeneous dispersal rates across species where $\sigma_d = 0.5$ are the black and green lines, while the scenarios with homogeneous dispersal rates across species where $\sigma_d = 0.01$ are the red and blue lines. A description of the scenarios is provided in Figure 1. Fixed parameters are as follows: $M = 30; N = 20; C = 0.2; m = -1;\ \mu_1 = 0;\ \sigma_1 = 0.05;\ \mu_2 = 0;\ \sigma_2 = 0.05;\ S = M + N;\ n = 10$.
